# The role of inflammatory cytokines in anemia and gastrointestinal mucosal injury induced by foot electric stimulation

**DOI:** 10.1038/s41598-021-82604-7

**Published:** 2021-02-04

**Authors:** Fangcheng Fan, Yangwen Ai, Ting Sun, Shuran Li, Hua Liu, Xiaojie Shi, Ziqian Zhang, Qingshan Liu, Yong Cheng

**Affiliations:** 1 Key Laboratory of Ethnomedicine of Ministry of Education, School of Pharmacy, Center On Translational Neuroscience, College of Life and Environmental Sciences, Minzu University of China, No. 27, South Street of Zhongguancun, Haidian District, Beijing, 100081 China; 2 Institute of Materia Medica, Chinese Academy of Medical Sciences and Peking Union Medical College, Beijing, 100032 China

**Keywords:** Stress and resilience, Upper gastrointestinal bleeding

## Abstract

Foot electrical stimulation (FES) has been considered as a classic stressor that can disturb homeostasis. Acute anemia was observed in the model induced by FES. The aim of this study was to explore the role of inflammatory cytokines underlying the acute anemia and gastrointestinal (GI) mucosal injury in the FES. Twenty-four male Kunming mice (20 ± 2 g) were randomly divided into control group and experimental group. The mice were placed in a footshock chamber that can generate 0.5 mA electrical impulse periodically for 0.5 h. After the process, red blood cell count, hemoglobin concentration and hematocrit, the levels of corticotropin releasing hormone (CRH) in serum and hypothalamus, and adrenocorticotropic hormone (ACTH) in serum and pituitary were detected separately. In addition, we investigated the expressions of inflammatory cytokines (IL-1, IL-6, TNF-α, iNOS, and IL-10) in the hypothalamus and duodenum by Polymerase Chain Reaction (PCR). Results showed that this FES model induced anemia, increased CRH and ACTH activity in the serum after the FES. Moreover, the expressions of IL-1β, IL-6, TNF-α, and iNOS were significantly increased following the process, while IL-10 was not activated. These findings suggest that anemia, the inflammatory cytokines in the hypothalamus and duodenum of the mice in the model induced by FES is closely related to GI mucosal injury/bleeding. Taken together, these results underscore the importance of anemia, GI mucosal injury/bleeding and stress, future studies would be needed to translate these findings into the benefit of affected patients.

## Introduction

Stress is defined as a non-specific response of the body to the noxious stimulus. Physiological and psychological stressors threaten the homeostasis of the body^1^. In recent years, foot electrical stimulation (FES) has been considered as a physical stressor that can lead to depression, disturb homeostasis, and affect the actions of many hormones in the body^2, 3^.

FES is an intense stressful procedure, mice experience an aversive procedure consisting in an electric footshock may induce depressive behaviors. This stress-related pathology such as depression is associated with profound disturbances of the hypothalamus–pituitary–adrenal (HPA) axis^4, 5^. The HPA axis is a neuroendocrine system that plays a key role in stress response^6^. Corticotropin releasing hormone (CRH), which is released from the paraventricular nucleus of the hypothalamus and response to many somatic stimuli, such as inflammation and hunger, is involved in the activation of HPA axis in the brain^7, 8^. The activation of CRH regulates the secretion of adrenocorticotropic hormone (ACTH) into the peripheral circulation, and then ACTH induces the secretion of glucocorticoids^9^. CRH is associated with gastric secretion, colonic motility, and gastric pH level^10^. The reduction of CRH and ATCH promoted jejunal contraction and gut motility by increasing Ghrelin^11^. It is well known that the activation of HPA axis is closely related to the disturbance of gastrointestinal (GI) hormones.

Cytokines are the regulatory proteins secreted by white blood cells and various other cells. They have numerous effects on immune system and inflammatory responses^12^. During immune responses, activated macrophages, T cells, and natural killer cells produce proinflammatory cytokines, which in turn regulate the inflammatory response system (IRS). Then IRS activates HPA axis, most notably CRH and ACTH, stimulating the secretion of catecholamines^13^. A previous study showed that proinflammatory cytokines, such as IL-1β, IL-6, and tumor necrosis factor-α (TNF-α), increased the release of CRH, suggesting the stimulation of HPA axis^14^. The activation of iNOS in macrophages is regulated by cellular receptor molecules, such as Toll-like receptors and CD14, which play a critical role in the pro-inflammatory response of monocytes and macrophages via NF-κB pathway^15^. The binding of IL-1β to TNF-α is an important mediator of iNOS, regulating the mRNA expression of iNOS^16^. Interleukin-10 (IL-10) is a key anti-inflammatory cytokine responsible for intestinal immune homeostasis, mainly through the inhibition of pro-inflammatory cytokines^17^. When the intestinal immune homeostasis is disturbed by stress, macrophages and T lymphocytes are activated to release pro-inflammatory cytokines, such as IL-1β and TNF-α^18^. Recently, pro- and anti-inflammatory cytokines have been used as biomarkers of oxidative stress^19, 20^.

Potent neutrophil-activated chemokines, such as IL-1β, IL-6, TNF-α, play crucial roles in acute gastroenteritis^21^. The intestinal endotoxin induces intestinal barrier injury and activates innate immunity to produce pro-inflammatory cytokines including IL-1β, IL-6, TNF-α, and iNOS. These plasma proteins destroy the intestinal epithelial barrier and increase the intestinal permeability^22–24^. IL-10 is an anti-inflammatory cytokine that plays an important role in the protection of epithelial integrity and the regulation of mucosal immune system in small intestine^25^.

Recent studies have shown that FES is a novel acute model of stress. However, it remains unclear whether inflammatory cytokines participate in FES-induced GI mucosal injury and bleeding. Therefore, this study aimed to explore the effects of this model on red blood cell (RBC) count, hemoglobin concentration and hematocrit, the levels of CRH in serum and hypothalamus, and ACTH in serum and pituitary. In addition, we examined the expressions of pro- and anti-inflammatory cytokines, including IL-1, IL-6, TNF-α, iNOS, and IL-10, in the hypothalamus and duodenum.

## Materials and methods

### Chemicals and reagents

Hematoxylin–Eosin (H&E) staining was purchased from Beijing Dingguo Biotechnology Development Center (Beijing, China). Mouse ACTH and CRH ELLISA Kit were performed by Elabscience Biotechnology Co., Ltd (Wuhan, China). The oligonucleotide primer pairs were synthesized by the Sangon Biotech (Beijing, China). Fecal occult blood kit was purchased from Beijing Leagene Biotechnology Co., Ltd (Beijing, China).

### Animals

Twenty-four male Kunming mice (20 ± 2 g) were purchased from Fangyuanyuan breeding Farm (Beijing, China) and housed in an environment-controlled room (22 ± 2 °C, natural light/dark cycle) with free access to water and food. All animals were acclimated in a single room for at least one week. Mice were fasted for 24 h with *ad lib* access to water before experiments.

### Protocols

A footshock chamber was designed to generate 0.5 mA electrical impulse by the Institute of Material Medica, Chinese Academy of Medical Science. This instrument was used to generate an animal model of depression by low voltage electricity. Mice were randomly divided into two groups: the control group and the experimental group. The experimental group was subjected to the instrument for 0.5 h, and the control mice were placed into the instrument without electric stimulation. The experiments were performed between 8:00 and 13:00 to eliminate the effect of circadian rhythm on the experiment. In this research, all experiments were conducted according to the guidelines of National Institutions of Health for the Use and Care of Lab Animals and took following to the Minzu University of China’s Ethics Review Board.

### Fecal occult blood test

Fecal occult blood tests use the presence of the peroxidase activity of hemoglobin, which oxidizes substances o-tolidine and induces a color change. The mice were allowed free access to water and food without animal blood, meat, and other special drugs rich in chlorophyll and iron for 3 days before the test. After fasted for 24 h with *ad lib* access to water, the mice were exposure to the electric stimulation. Three hours after process, fecal samples were collected and placed on a white porcelain plate. After 0.1 mL *o*-tolidine solution were added to the feces, and then 0.1 mL oxidant were dropped. The test proved positive if the reagent changed gradually to green after 2 min.

### The measurement of RBC count, hemoglobin and hematocrit

After the FES, the blood was taken from tail vein. A volume of 20 µL whole blood was used for the measurement of RBC count, hemoglobin concentration and hematocrit using a Hematology analyzer (MEK-6318, Japan).

### Macroscopic and histological analysis of duodenum and stomach

Mice were sacrificed after blood collection, and the stomachs and duodenums were harvested and fixed. The GI inner wall was identified with anatomical lens after fixed with 4% paraformaldehyde for 30 min. The scale of GI mucosal injury was determined by anatomical microscope. The gastric erosion index (EI) was measured by the Guth method^26^. The score is calculated according to the length of the injury. The length ≤ 1 mm is 1 point; between 1 and 2 mm is 2 points; and the rest is calculated in turn. When the length is greater than 1 mm, the score doubles. In a mouse, the cumulative score of all lesions was defined as the EI of the mouse.

After the morphological observation of the duodenum and stomach, the samples were fixed with 4% paraformaldehyde, dehydrated, embedded in paraffin, and stained with H&E. Duodenal samples were collected from the same site and frozen at − 80 ℃ for further analysis.

### The measurement of CRH and ACTH

The concentrations of CRH levels in hypothalamus, ACTH levels in pituitary, CRH and ACTH levels in serum were analyzed using an enzyme-linked immunosorbent (ELISA) kit. Hypothalamus and pituitary tissues were weighed and minced into small pieces, which were homogenized in 10 mg: 90 µL PBS with protease inhibitor. All procedure was carried out according to the instructions of the kit. The blood samples were centrifuged for 15 min at 3000 rpm and the serum was collected for the detection of CRH and ACTH levels (50 µL for each measurement). The working solution was added into the 96-well Costar plate and the ELISA reaction was detected at the corresponding wavelength using a microplate reader.

### RNA isolation

The total RNAs of the hypothalamus and duodenum were isolated using TRIzol Reagent. In detail, 50 mg organization was homogenized and transferred to 1 mL TRIzol lysis. After adding 700 µL 75% ethanol, the total RNAs were dissolved by 50 µL water. The concentration and purity of the RNAs were quantitated by Quickdrop (Molecular Devices cat., USA). The reverse transcription of the RNAs was amplified by polymerase chain reaction (PCR). For cDNA synthesis, 10 ng cDNA was incubated with 0.2 µM primers and 10 µL 2 × RealStar Green Fast Mixture (Genestar, China). The reaction conditions were as follows: 2 min at 95 °C, then 95 °C for 15 s, followed by 41 cycles at 60 °C for 30 s. The oligonucleotide primer pairs were shown in Table 1. The relative mRNA levels were analyzed according to the 2 − ΔΔCt method^27^.

**Table 1:** Gene specific primers for PCR.

Gene	Orientation accession	Number sequence
β-Actin	Forward	AGATCAAGATCATTGCTCCTCCT
	Reverse	CTCAGTAACAGTCCGCCTAGAA
IL-1β	Forward	GCAACTGTTCCTGAACTCAACT
	Reverse	ATCTTTTGGGGTCCGTCAACT
IL-6	Forward	TAGTCCTTCCTACCCCAATTTCC
	Reverse	TTGGTCCTTAGCCACTCCTTC
IL-10	Forward	GCTCTTACTGACTGGCATGAG
	Reverse	CGCAGCTCTAGGAGCATGTG
TNF-α	Forward	GACGTGGAACTGGCAGAAGAG
	Reverse	TTGGTGGTTTGTGAGTGTGAG
iNOS	Forward	GTTCTCAGCCCAACAATACAAGA
	Reverse	GTGGACGGGTCGATGTCAC

### Statistical analysis

All the results were calculated using a t-test or a repeated measure two-way analysis of variance (ANOVA). Results were reported as mean values ± SD. *P* < 0.05 was considered to be significant.

## Results

### GI mucosal injury and bleeding during FES

**Figure 1:**
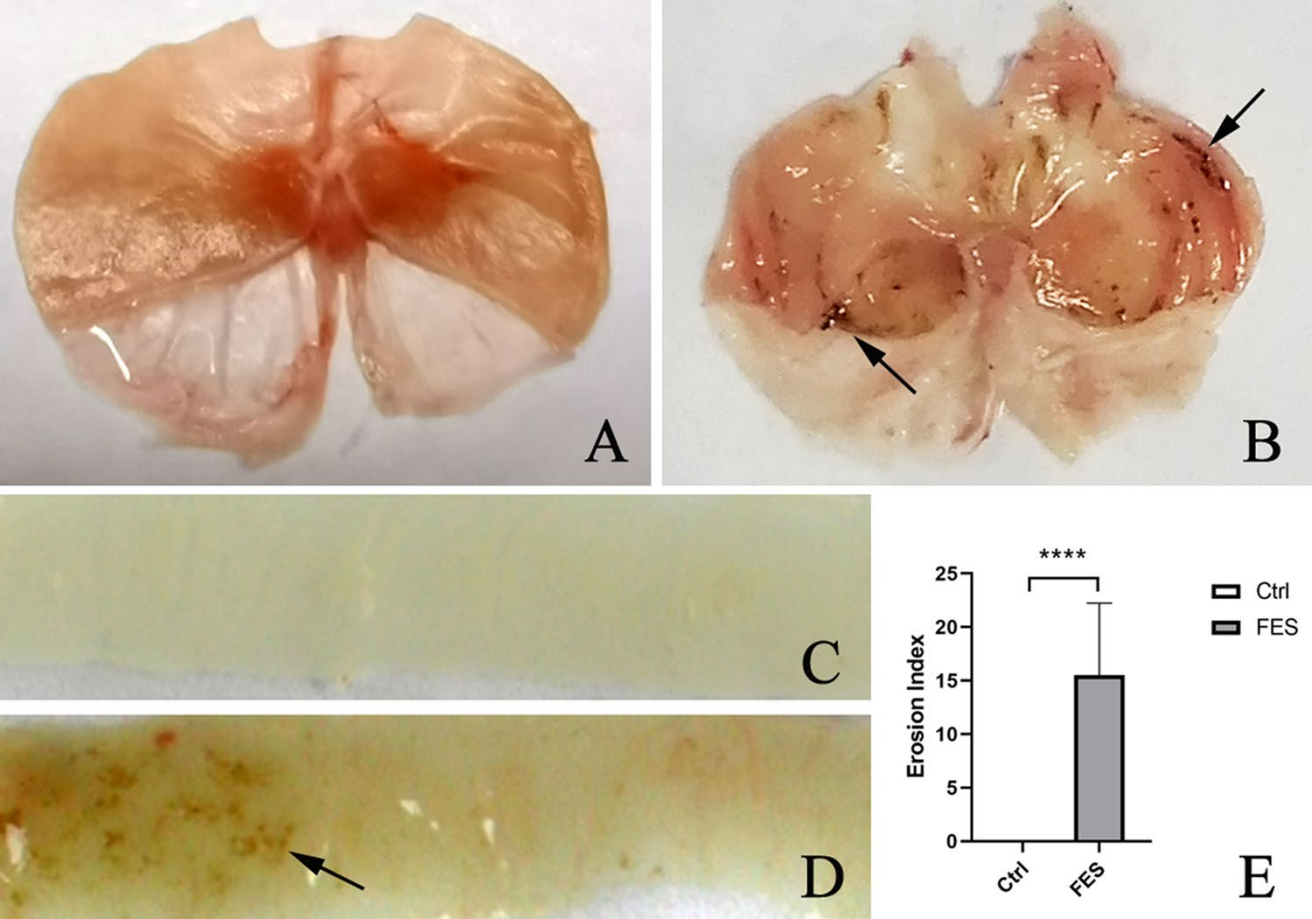
The effects of FES on duodenum and stomach: (**A**) Stomach of Pre- FES, (**B**) Stomach of After- FES, (**C**) Duodenum of Pre- FES, (**D**) Duodenum of After- FES, (**E**) Effects of FES on gastric mucosal damage. All data are presented as mean ± SD (n = 12). ^****^P < 0.001 versus control group.

**Figure 2:**
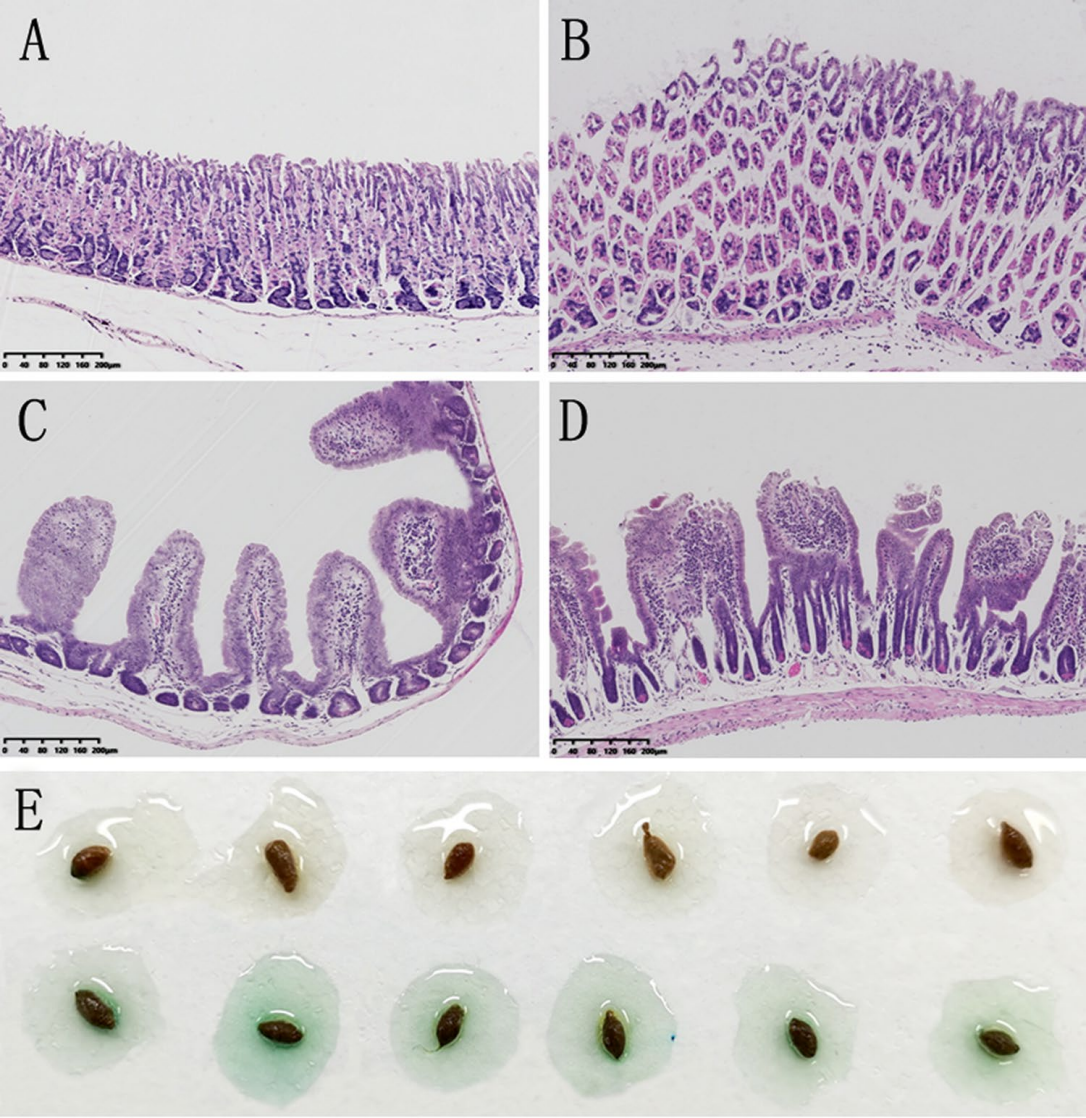
HE staining of duodenum and stomach: (**A**) Duodenum of Pre- FES (×10), (**B**) Duodenum of After- FES (×10), (**C**) Stomach of Pre- FES (× 10), (**D**) Stomach of After- FES (×10), (**E**) Fecal occult blood test.

The effects of FES on the morphological changes of stomach and duodenum are shown in Fig. 1. The gastric mucosa and intestinal wall in the control group were smooth, uniform and translucent. Mice with FES treatment showed dark brown areas on the surface and the lesions, and there was a significant difference compared to the control group (Fig. 1E). Histological analysis with H&E staining also matched with the macroscopic observations (Fig. 2). Outcomes of H&E staining showed gastric and duodenal mucosal epithelia defect, epithelial cell loss and lamina propria mucosal erosions as well as inflammatory cell infiltration in the model group. In addition, the fecal occult blood test was positive (Fig. 2E). These findings suggested that FES induced GI mucosal injury and bleeding in mice.

### Determination of RBC count, hemoglobin, hematocrit

**Figure 3:**
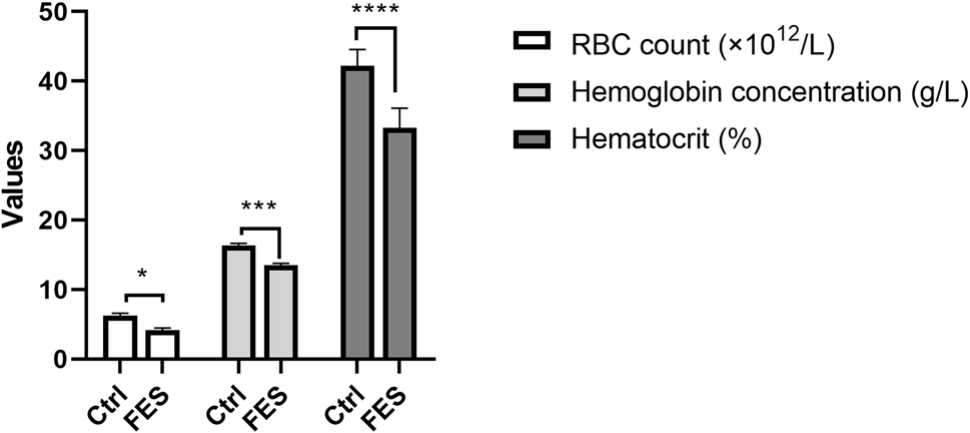
The effects of FES on RBC, hemoglobin and hematocrit. All data are presented as mean ± SD (n = 12). *P < 0.05 versus control group, ***P < 0.005 vs. control group, ****P < 0.001 versus control group.

The RBC count, the concentration of hemoglobin and hematocrit of the experimental group were significantly lower than those in the control group, indicating that mice were anemia after the process (Fig. 3).

### Effects of FES on levels of CRH and ACTH

**Figure 4:**
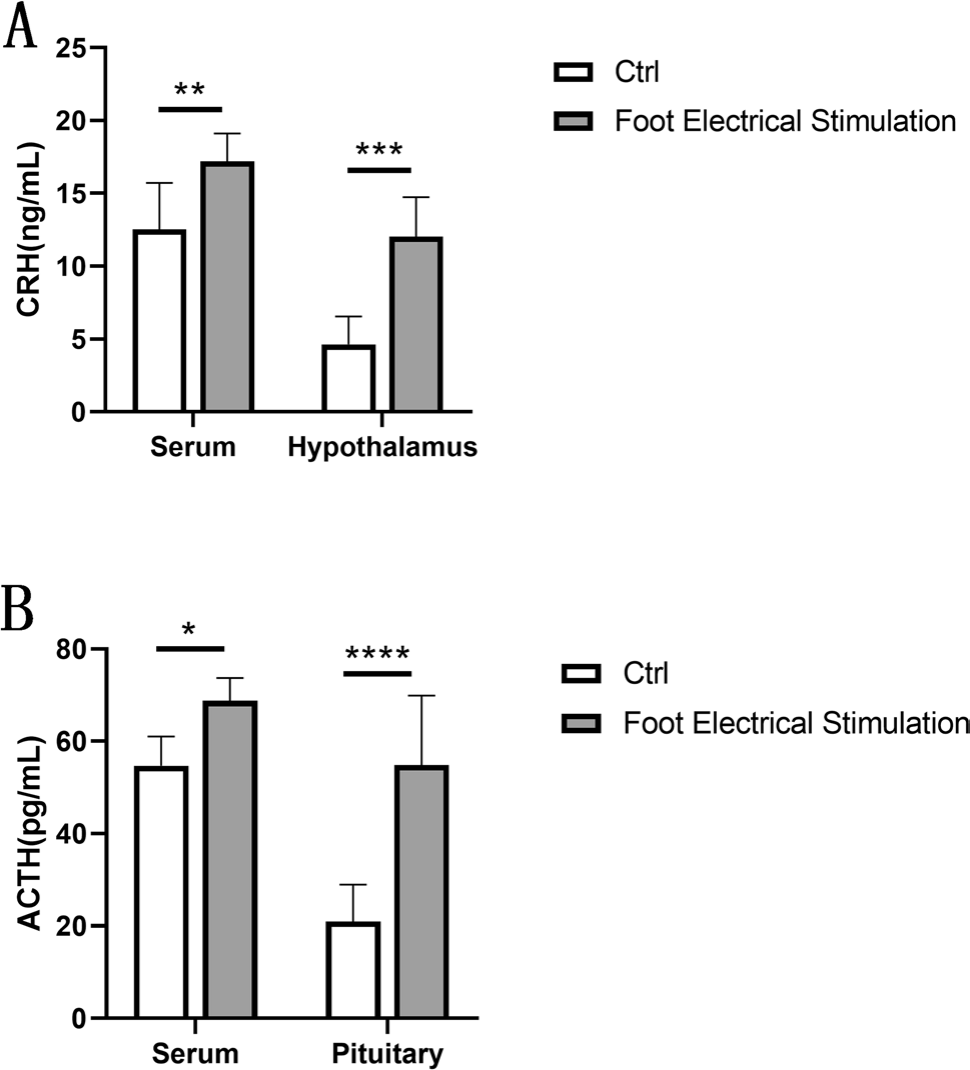
(**A**) The effects of FES on CRH in serum and the Hypothalamus. (**B**) The effects of FES on ACTH in serum and the pituitary. All data are presented as mean ± SD (n = 12). *P < 0.05 versus control group, **P < 0.01 versus control group, ***P < 0.005 versus control group, ****P < 0.001 versus control group.

As shown in Fig. 4, the levels of CRH were significantly increased in the hypothalamus and serum, and the levels of ACTH were significantly increased in the pituitary and serum compared to the controls, suggesting an increase in CRH and ACTH levels after the stress. These data implied that CRH and ACTH might be involved in the stress process.

### Effects of FES on the expressions of cytokines in the hypothalamus

**Figure 5:**
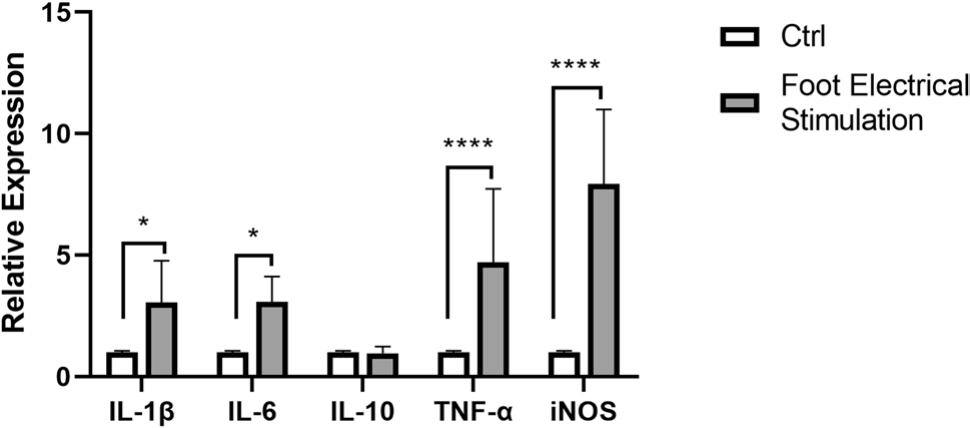
The levels of IL-1β, IL-6, IL-10, TNF-α, and iNOS gene in hypothalamus of control and experimental group were detected by PCR. All data are presented as mean ± SD (n = 12). *P < 0.05 versus control group, ****P < 0.001 versus control group.

To determine the expression levels of IL-lβ, IL-6, IL-10, TNF-α, and iNOS, the total RNAs were isolated from hypothalamus samples. As shown in Fig. 5, the expressions of cytokine genes were relatively low without stress. After FES, the levels of Il-1β, IL-6, TNF-α, and iNOS were largely increased. No significant difference was observed on the change of IL-10 expression before and after the stress. iNOS gene showed a stronger response to the stress (Fig. 5). The above results suggested that these cytokines in the hypothalamus participated in the FES.

### Effects of FES on cytokine gene expressions in the duodenum

**Figure 6:**
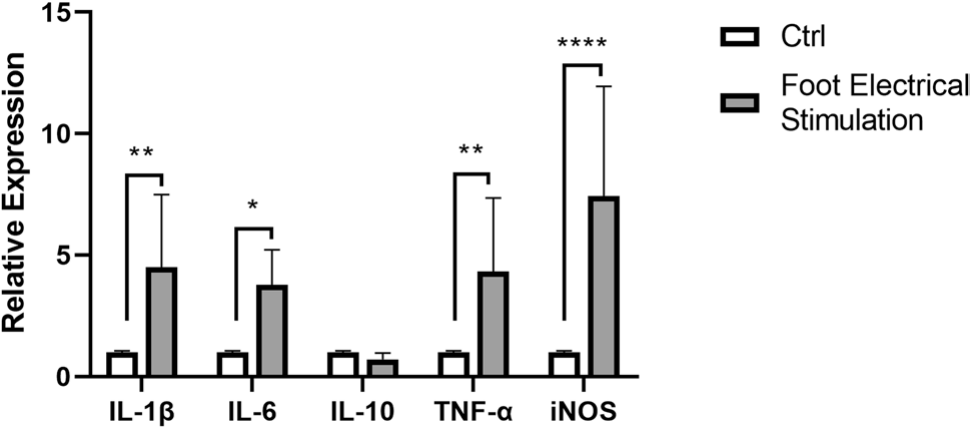
The levels of IL-1β, IL-6, IL-10, TNF-α, and iNOS gene in duodenum of control and experimental group were detected by PCR. All data are presented as mean ± SD (n = 12). *P < 0.05 versus control group, **P < 0.01 versus control group, ****P < 0.001 versus control group.

Two-way ANOVA analysis showed that FES significantly affected the expressions of IL-1β, IL-6, IL-10, TNF-α, and iNOS in the duodenum. The levels of cytokines in the experiment group were significantly increased compared to the controls (Fig. 6).

## Discussion

In this study, our results confirmed that FES can affect the levels of CRH, ACTH, anemia and GI mucosal injury and bleeding in the FES group. Moreover, pro- and anti-inflammatory cytokines (IL-1β, IL-6, IL-10, TNF-α, and iNOS) may play a role in the initiation of GI mucosal injury/bleeding induced by FES.

To investigate whether the model was related to GI mucosal injury and bleeding, we observed the morphological changes of gastric and duodenal mucosa after stress. Acute GI mucosal injury and bleeding following stress indicated that GI Mucosal Injury was involved in this process. It was consistent with the data obtained from animal models and patients, showing that GI mucosal cell damage was induced by GI Mucosal Injury and ischemia^28^. The decreased RBC count, hemoglobin concentration and hematocrit are a marker of anemia^29, 30^. Our results suggested that anemia was induced by the FES.

Physiological systems of the body respond to stress stimuli to promote defense and survival. In the present study, the animal model was considered as a special stressor that might induce GI mucosal injury/bleeding. The HPA axis is an important regulatory system for regulating stress response. During stress exposure, CRH is released from the hypothalamus and regulates the secretion of ACTH, and then ACTH induces the secretion of glucocorticoids^31, 32^. In our study, we observed a significant increase in serum CRH and ACTH levels in model animals. In addition, the stress increased the levels of CRH in hypothalamus and ACTH in the pituitary.

Previous studies have found that CRH and ACTH are associated with immune system and inflammatory responses^33, 34^. As a result, we explored the effects of pro- and anti-inflammatory cytokines (IL-1β, IL-6, TNF-α, iNOS, and IL-10) in the hypothalamus. The results showed that the expressions of pro-inflammatory cytokines (IL-1β, IL-6, TNF-α, and iNOS) after stress were significantly increased compared to the control group. It has been found that the level of IL-1β is significantly increased in the hypothalamus of rat with restraint stress^35^. In addition, IL-6 and TNF-α produce corticotropin-releasing GABA by stimulating HPA axis^36^. Hypothalamic paraventricular nucleus repairs colon injury by regulating the expressions of TNF-α and IL-1β^37^. Clinical studies found that the activity of NOS in patients with celiac disease was higher than that in other patients^38^. Animal studies showed that IL-1β was the key mediator of nitric oxide after endotoxin exposure. It promoted microglia to produce vasodilator and neuromodulator nitric oxide by increasing the biosynthesis of iNOS^39^. Our findings were in line with previous studies, which suggested a mutual activation relationship of pro-inflammatory cytokines. Our data on the expression of IL-10 was also consistent with that in the excessive eccentric FES model, in which the levels of IL-1β and IL-6 were increased, while the expression of IL-10 was decreased^40^.

Furthermore, we measured the expressions of well-known inflammatory markers in the duodenum. Pro-inflammatory cytokines (IL-1β, IL-6, TNF-α, and iNOS) were significantly increased as compared to the controls after stress. Recent evidence has shown that IL-1β increases the intestinal permeability^41^. In intestinal cells, IL-1β partially increased the intestinal permeability by reducing the expression and redistribution of occludin^22^. IL-6 is mainly distributed in intestinal monocytes and the activation of IL-6 promotes the proliferation and repair of intestinal epithelium after injury^42^. It has been reported that TNF-α induces the apoptosis and inflammation of intestinal epithelial cells, and damages the intestinal mucosal barrier. TNF-α plays an essential role in regulating GI diseases and its expression in intestinal cells is closely correlated with intestinal barrier defect^43, 44^. NOS produces excessive nitric oxide (NO) in the progression of various intestinal inflammatory diseases. The synthesis of NO by iNOS is associated with a variety of pathophysiological processes, including inflammation. Clinical data showed that the activity of iNOS in duodenal epithelial cells was increased in patients with celiac disease^45, 46^. Consistently, we found that the pro-inflammatory cytokines (IL-1β, IL-6, TNF-α, and iNOS) in the duodenum were activated, resulting in GI mucosal injury/bleeding and the activation of immune responses in mice. IL-10 is a key anti-inflammatory cytokine produced by intestinal macrophages^38^. As shown in an in vitro study, IL-10 exhibited opposite effects on cellular functions when compared to TNF-α, IL-1β, and IL-6^43^. Here, we found that IL-10 was not activated in the animal model, which was consistent with previous findings.

**Figure 7:**
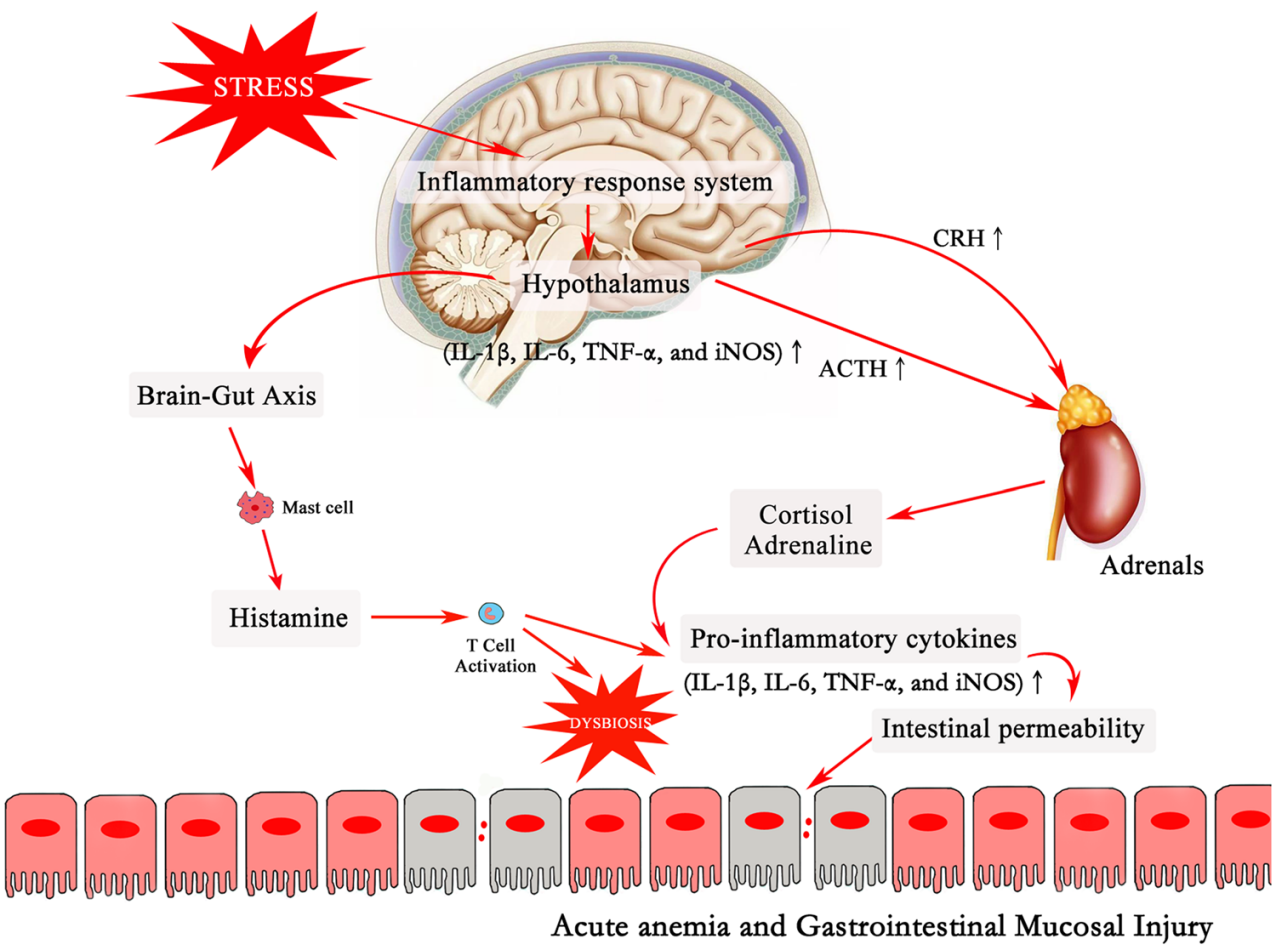
The mechanism of stress affecting brain, GI mucosa and GI permeability through inflammatory response system.

As shown in Fig. 7, IRS activated the hypothalamic CRH system, resulting in the secretion of ACTH into the peripheral circulation and the induction of glucocorticoids. Then the brain-gut axis activates mucosal mast cells, increases the expressions of pro-inflammatory cytokines, and promotes the activity of endocrine gland^9, 47^. The intestinal homeostasis is maintained by the neural connections through the brain-gut axis, together with the regulation of reactive oxygen metabolites and pro-inflammatory cytokines, such as IL-1 β, TNF-α, and IL-6. A study of intestinal cells found that the overexpression of pro-inflammatory cytokines increased GI permeability and induced GI bleeding^48^. In this study, the expressions of pro-inflammatory cytokines (IL-1β, IL-6, TNF-α, and iNOS) in hypothalamus and duodenum were significantly increased following GI mucosal injury. Our model of FES induced-GI mucosal injury/bleeding was consistent with the results of previous studies.

## Conclusion

In conclusion, our results indicate that GI mucosal injury induced by FES is related to anemia. A correlation was found between GI mucosal injury/bleeding and the expressions of pro-inflammatory cytokines (IL-1β, IL-6, TNF-α, and iNOS) in the hypothalamus and duodenum. This is the first study to show that the activation of IRS is closely associated with anemia and GI mucosal injury/bleeding induced by FES. This model may be used for future investigations on depression stress-induced anemia and GI mucosal injury/bleeding.
